# Therapeutic Potential of Marine Bioactive Peptides against Human Immunodeficiency Virus: Recent Evidence, Challenges, and Future Trends

**DOI:** 10.3390/md20080477

**Published:** 2022-07-25

**Authors:** Jameel Mohammed Al-Khayri, Waqas Asghar, Sipper Khan, Aqsa Akhtar, Haris Ayub, Nauman Khalid, Fatima Mohammed Alessa, Muneera Qassim Al-Mssallem, Adel Abdel-Sabour Rezk, Wael Fathi Shehata

**Affiliations:** 1 Department of Plant Biotechnology, College of Agriculture and Food Sciences, King Faisal University, Al-Ahsa 31982, Saudi Arabia; arazk@kfu.edu.sa (A.A.-S.R.); wshehata@kfu.edu.sa (W.F.S.); 2 School of Food and Agricultural Sciences, University of Management and Technology, Lahore 54770, Pakistan; waqas.asghar@umt.edu.pk (W.A.); sipperkhan@gmail.com (S.K.); aqsa.akhtar@umt.edu.pk (A.A.); haris.ayub@umt.edu.pk (H.A.); 3 Department of Food Science and Nutrition, College of Agriculture and Food Sciences, King Faisal University, Al-Ahsa 31982, Saudi Arabia; falissa@kfu.edu.sa (F.M.A.); mmssallem@kfu.edu.sa (M.Q.A.-M.)

**Keywords:** bioactive peptides, anti-HIV, marine organisms, antiretroviral agents, drugs

## Abstract

Acquired immunodeficiency syndrome (AIDS) is a chronic and potentially fatal ailment caused by the human immunodeficiency virus (HIV) and remains a major health problem worldwide. In recent years, the research focus has shifted to a greater emphasis on complementing treatment regimens involving conventional antiretroviral (ARV) drug therapies with novel lead structures isolated from various marine organisms that have the potential to be utilized as therapeutics for the management of HIV-AIDS. The present review summarizes the recent developments regarding bioactive peptides sourced from various marine organisms. This includes a discussion encompassing the potential of these novel marine bioactive peptides with regard to antiretroviral activities against HIV, preparation, purification, and processing techniques, in addition to insight into the future trends with an emphasis on the potential of exploration and evaluation of novel peptides to be developed into effective antiretroviral drugs.

## 1. Introduction

Research in recent decades has highlighted the potential of natural bioactive compounds for the clinical management of various diseases, such as acquired immunodeficiency syndrome (AIDS), caused by the human immunodeficiency virus (HIV) [1]. Marine bioactive peptides (MBAPs) that are present in many marine species, including fish, sponges, cyanobacteria, fungi, ascidians, seaweeds, and mollusks, have gained attention for their health-promoting benefits. MBAPs obtained from marine species have ameliorating potential against many health conditions, such as hypertension, diabetes, obesity, HIV, cancer, oxidation, and inflammation. Various research studies have indicated that MBAPs can be utilized for the treatment of HIV in conjunction with pharmaceuticals and functional foods due to their antitherapeutic effect [2].

**Figure 1:**
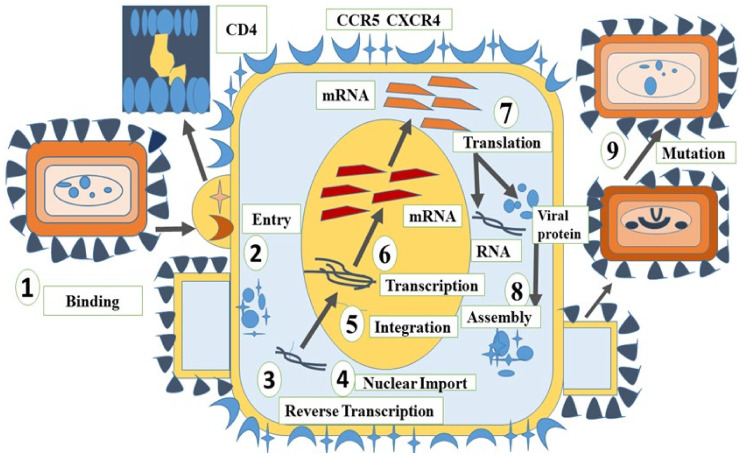
Depiction of the life cycle of Human Immunodeficiency Virus (HIV). (**1**) Binding, (**2**) viral entry, (**3**) reverse transcription, (**4**) nuclear import, (**5**) integration, (**6**) transcription, (**7**) translation, (**8**) assembly, and (**9**) mutation.

HIV is a chronic and potentially life-threatening condition, and as per the Joint United Nations Programme on HIV/AIDS (UNAIDS) report of 2020, over 37.7 million people were living with HIV globally [3]. During the life cycle of the disease, the virus attaches itself to the host cells, followed by the cluster of differentiation 4 (CD4) receptor binding, followed by co-receptor binding that ultimately leads to the fusion of the viral and host cell membranes [4,5]. This attachment phase involves the initial interaction of the envelope glycoproteins (Env) to the CD4 receptor followed by the binding of the phenylalanine 43 (Phe43) of CD4 to the gp120 residues inside the gp120 Phe43 cavity, consequently triggering major conformational changes in the viral envelope section, exposing the conserved gp120 region, and therefore facilitating the attachment of chemokine co-receptors, C-C chemokine receptor type 5 (CCR5), and C-X-C Motif Chemokine Receptor 4 (CXCR4) [6]. Subunits of gp41 unwind further, resulting in a six-helix coil formation that fuses gp41 into the cell membrane, thereby permitting the entry of viral genetic makeup into the cytosol [7]. After the entry of genetic material into the cytoplasm, it undergoes reverse transcription with the aid of reverse transcriptase (RT), resulting in the formation of HIV-1 DNA, which later serves as the template for the synthesis of mRNA transcripts of HIV-1 [8]. Enzyme integrase effectively integrates the viral DNA into the host cell genome [9]. After the completion of the translation process in the cytoplasm, new virus infectious particles (virions) are formed, which also undergo trimming inside the capsid and may infect the targeted cells, responding as a mature virus [10] as depicted in Figure 1.

Various research studies highlight the potential of MBAPs against the replication of HIV [11,12,13,14]. The current review considers the existing knowledge regarding the activity of MBAPs against HIV by exploring databases such as PubMed, ScienceDirect, SciFinder-n, and Embase, and using keyword combinations including bioactive peptides, marine bioactive peptides, anti-HIV, marine organisms, and antiretroviral agents. We selected 450 articles due to relevancy with the current theme, and after scrutinization, 165 were selected and reviewed.

## 2. Marine Bioactive Peptides (MBAPs)

Bioactive peptides (BAPs) from various marine organisms demonstrate extensive structural diversity and have gained popularity for the development of products in pharmaceutical, cosmeceutical [15,16,17], and nutraceutical industries [18,19,20]. MBAPs are largely involved in vital mechanisms such as growth, reproduction, and homeostasis, which are significant for the survival of living organisms [21]. BAPs are composed of between 3–20 diverse types of residual amino acids with functionality largely dependent on the sequential composition of these amino acids [22]. The BAPs with lower molecular weights assimilate through the intestinal wall without any hindrance, thereby increasing the functional and biological potential of BAPs as compared to other bioactive agents [23,24].

Owing to high membrane permeability characteristics, MBAPs exhibit a host of biological activities, including antimicrobial, antiviral [15,25,26,27], antibacterial [28,29,30,31], antifungal [32,33,34], cytotoxic [35], neurotoxic [36], anticoagulant [37,38], antidiabetic [25], antifreezing [39,40], antioxidant [41,42,43,44,45], immune-modulating [46,47], angiotensin I-converting enzyme (ACE)-inhibiting [48,49,50,51], and endotoxin binding capabilities [52,53,54]. The recent discovery of linear and cyclic peptides with novel chemical structural attributes in marine organisms [55] is of considerable significance. These peptides have exhibited strong ion channel-specific blocking potential along with other pathological and pharmacological prospects [56,57,58,59].

One of the primary challenges faced in the production of peptides from algae is the variability of protein content with the change in locality, temperature, and season. Furthermore, the extraction process poses significant complications, as the macroalgal cell wall is complex, rigid, is composed of intricate covalently bound polysaccharides (e.g., alginates), polyphenols, and proteins aligned in a complex covalently bonded structure, which are a major obstacle in the effective extraction and digestibility of BAPs [24,60,61].

Recently, increasing scientific evidence indicates that various hydrolyzed peptides isolated from marine organisms and fishery wastes (skin, intestines, head, and fins) have the potential to contribute to health-promoting effects and the prevention of many chronic diseases [62]. In the context of therapeutic potential, MBAPs offer significant health benefits with respect to complementing treatment regimens involving conventional antiretroviral (ARV) drug therapies for the treatment of chronic disease conditions [63,64].

As discussed above, MBAPs owing to their bioactive characteristics could be used in the development of potential nutraceuticals, functional foods, and food supplements [18]. Out of hundreds of peptides isolated from marine organisms, only a few have been approved for clinical trials and even fewer have been able to reach the market. Additionally, for many, the trials have been discontinued or terminated due to the lack of objective responses, effectiveness, or in worst-case scenarios, adverse outcomes in patients due to poor clinical trials [65,66].

### 2.1. Techniques Used for the Commercial Preparation and Purification of MBAPs

**Figure 2:**
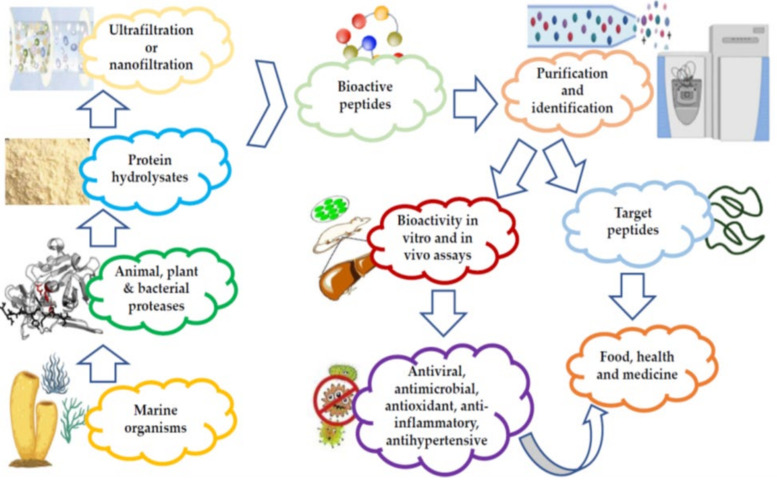
Preparation and purification of marine peptides.

MBAPs have been extracted from various sources, including fish, algae, crustaceans, and mollusks, in addition to various marine waste products, such as shells, substandard muscles, viscera, trimmings, and skins [67]. The BAPs are either procured from proteins during digestion and food processing, or are already present in the products prior to the processing operations [68]. The latter consist of both ribosomal bioactive peptides, in addition to non-ribosomal bioactive peptides (depsipeptides, cyclic peptides, and non-natural amino acid residues) [69]. Figure 2 is a schematic representation of the stages involved in the preparation and purification of marine peptides.

Different techniques have been used for the extraction of these peptides, such as organic solvents and acid/alkaline solutions. Extraction is followed by isoelectric precipitation, although, this results in the production of peptides with potentially adverse effects on the environment. Additionally, it is often accompanied by a high cost of production, leading to a longer purification process [14,70]. Recent research on antimicrobial marine peptides, or their fractions thereof, are reported to have a molecular weight <10 kDa and a high degree of antimicrobial activity [71]. Some of the techniques employed for the preparation and purification of MBAPs are listed in Table 1.

### 2.2. Marine Sponge-Derived Bioactive Peptides against HIV

Marine sponges are soft-structured, filter-feeding, bioactive component-rich, aquatic invertebrate parazoans from the phylum *Porifera*, and act as diversified habitats for numerous marine species [86]. Sponges contain diverse biomolecules of varying chemical and structural characteristics that exhibit various bioactive attributes [87]. This is proven by the fact that out of nine FDA-approved marine drugs, four are contributed by sponges [88]. Research reports suggest that many of the bioactive metabolites isolated from sponges are generated by their functional enzyme clusters and the microorganisms associated with these sponges [89].

Koshikamides from the sponges of *Theonella* sp. have been reported to exhibit anti-HIV activity. In this regard, relative to their linear counterparts, the cyclic koshikamides F and H inhibited HIV entry with IC_50_ values of 2.3 and 5.5 µM, respectively, when tested in a single round HIV-1 infectivity assay against a CCR5-using viral envelope [90,91]. Additionally, the peptides were evaluated for their cytotoxicity against various target cell lines, including a control kidney cell line (BSC-1), a human colon tumor cell line (HCT-116), and the target cell line TZM-bl. Favorably, neither koshikamide F nor koshikamide H exhibited cytotoxicity toward any of the target cell lines at these concentrations [90]. Although, koshikamide H was found to exhibit moderate cytotoxicity against the HCT-116 target cell line with an IC_50_ value of 10 µM [92]. The anti-HIV activity of the cyclic koshikamides F and H has been attributed to the presence of the 10 AA residue lactone ring in their structures, a characteristic absent in the linear analogs of these peptides [55]. Furthermore, koshikamide F has been proven to be slightly more potent in terms of anti-HIV activity, owing to the distinctive macrolactone conformation induced by the presence of the unsaturated pyrrolidinone residue Apdp [90].

Depsipeptides (also termed cyclodepsipeptides) with unique non-proteinogenic amino acid combinations inherently incorporated into their structures are isolated from various species of marine sponges and are of particular interest as powerful molecules aimed at drug development against HIV [88]. Callipeltin A (source: *Callipelta* sp. and *Latrunculia* sp.) and neamphamide A (source: *Neamphius huxleyi*) have also been proven to prevent the replication of HIV [93]. Callipeltin A, in an 3-(4,5-dimethylthiazol-2-yl)-2,5-diphenyltetrazolium bromide (MTT) cell viability assay, inhibited HIV-1-induced (Lai strain, X4 tropic) cytopathic effects in CEM4 lymphocytic cell lines at an EC_50_ value of 0.01 μg/mL, and a TC_50_ value of 0.29 μg/mL [94]. Additionally, the structural similarities to the potent antiviral family of compounds called didemnins might also imply that callipeltin A possesses anti-HIV activity [95]. Similarly, neamphamide A demonstrated HIV-inhibitory activity in a 2,3-Bis-(2-Methoxy-4-Nitro-5-Sulfophenyl)-2*H*-Tetrazolium-5-Carboxanilide (XTT) cell viability assay in a human T-cell line CEM-SS infected with HIV-1_RF_ at an IC_50_ value of 28 nM, and a TC_50_ value of 260 nM [94]. Furthermore, homophymine A, a depsiundecapeptide from a New Caledonian species of marine sponges, *Homophymia* sp. demonstrated cytoprotective activity against HIV infection. The anti-HIV activity was evaluated by an XTT cell viability assay in HIV-1-infected cells (IIIB strain). An assessment at seven days post-infection indicated that the compound inhibited HIV-1 infection production with an IC_50_ value of 75 nM [96,97]. Also, a TC_50_ value of 1.19 μM was recorded against host cells when direct cytotoxic measurements of homophymine A were undertaken [96,97].

Anti-HIV activity has also been recorded for mirabamides E–H derived from the marine sponges *Stelletta clavosa*, along with the already recognized mirabamides A–D isolated from the sponge *Siliquariaspongia mirabilis*. The activity of mirabamides was tested using HIV-1 neutralization assays against two viral strains, HXB2 (T-cell-tropic) and SF162 (macrophage-tropic), in addition to an HIV-1 envelope-mediated cell fusion assay to ascertain whether these peptides act to prevent the entry of the virus during the initial stages of the infection [94,98]. Mirabamides A, C, and D exhibited significant inhibitory activity against HXB2 infection of the TZM-bl host cells (involved in the expression of CXCR4, CCR5, and CD4) with IC_50_ values of 140 nM, 140 nM, and 190 nM, respectively, while mirabamide B was relatively less effective with an IC_50_ value of >50 μM [94,98]. Comparatively, mirabamides A, C, and D were relatively less potent towards SF162 (IC_50_ values of 400 nM, 1 μM, and 1 μM, respectively), while mirabamide B once again exhibited weak inhibitory activity toward SF162 [94,98]. As mirabamides A, C, and D have been found to inhibit HIV in both the aforementioned neutralization and fusion assays, it may be surmised that these peptides can act at the initial stages of HIV entry into the host cell, preventing viral entry and subsequent fusion [94,98]. Likewise, pseudotyped viruses with an enveloped HIV-1 strain when tested in single-round infectivity assays against the new cyclodepsipeptides mirabamides E–H (isolated from the hairy olives sponge *S. clavosa)*, in parallel with mirabamide C, exhibited significant antiviral replication activity in genital epithelial cells (expressing CCR5 and CD4 HIV-1 coreceptors) with IC_50_ values of 121, 62, 68, and 41 nM, for mirabamides E–H, respectively. Therefore, these compounds also seem to be inhibitors of viral entry during the initial stages of infection by virtue of their binding capabilities to the HIV-1 envelope glycoprotein for inhibition of viral fusion into the host cell membrane [99].

Along with the previously reported depsipeptides with HIV-inhibitory activity (such as mirabamides) from *S. clavosa*, two new depsipeptides, namely stellettapeptin A (EC_50_: 23 nmol/L) and stellettapeptin B (EC_50_: 27 nmol/L) were discovered, exhibiting potent HIV-inhibitory activities [88]. For this study, the anti-HIV activity of stellettapeptins was evaluated in an XTT-based cell viability assay by using the human T-cell line CEM-SS infected with HIV-1_RF_. After an incubation period of 6 days, the incubated compounds were found effective to inhibit the cytopathic effect of HIV-1. The toxicity of these depsipeptides against the host cells was observed with IC_50_ values of 367 and 373 nM, respectively [100].

Microspinosamide, a tridecadepsipeptide from the sponge *Sidonops microspinosa,* is another cyclic depsipeptide with an inhibitory effect against cytopathic action of HIV-1 infection in an in vitro XTT-based assay with an EC_50_ value of 0.2 μg/mL [101]. Similarly, theopapuamide B, isolated from the bacteria symbiosis sponges *Theonella swinhoei* and *S. mirabilis* exhibited anti-HIV activity in an in vitro single-round HIV-1 infectivity assay against HIV-1 SF162 envelope pseudotyped viruses with an IC_50_ value of 0.8 µg/mL [101]. Celebeside A from *S. mirabilis* has similarly been attributed with HIV-1 neutralization activity with an IC_50_ value of 1.9 µg/mL in a single-round infectivity assay [101].

**Figure 3:**
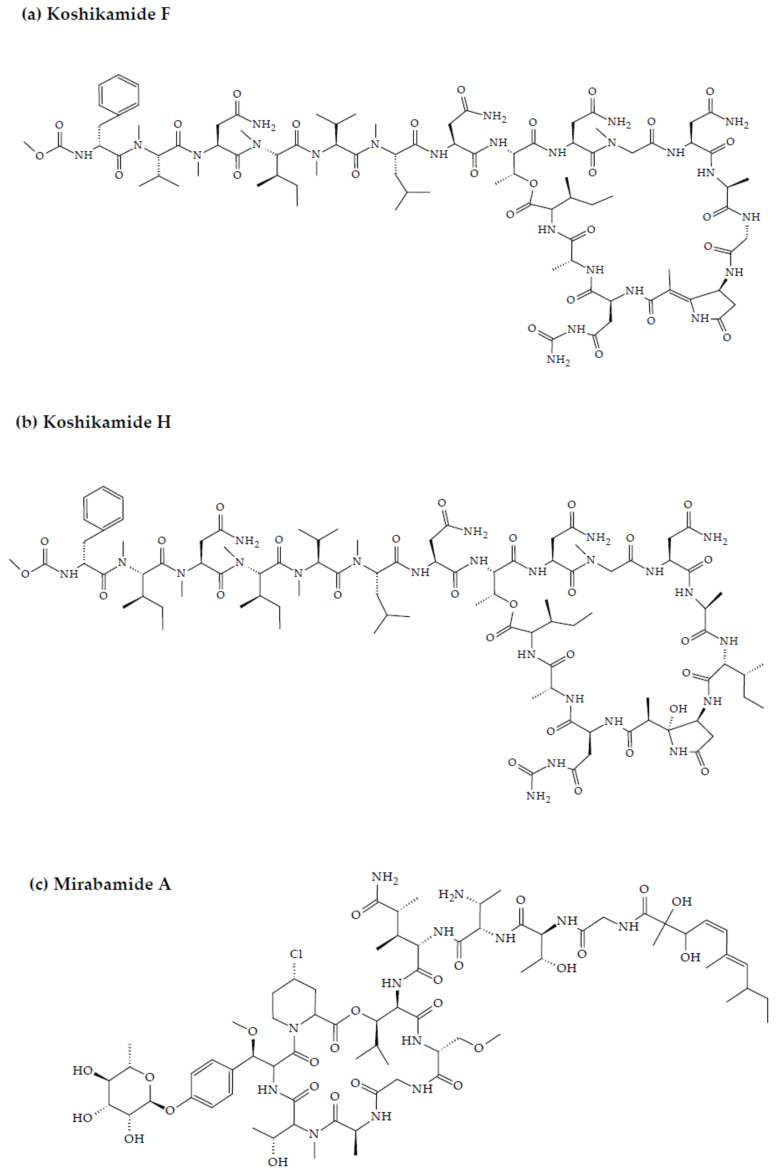

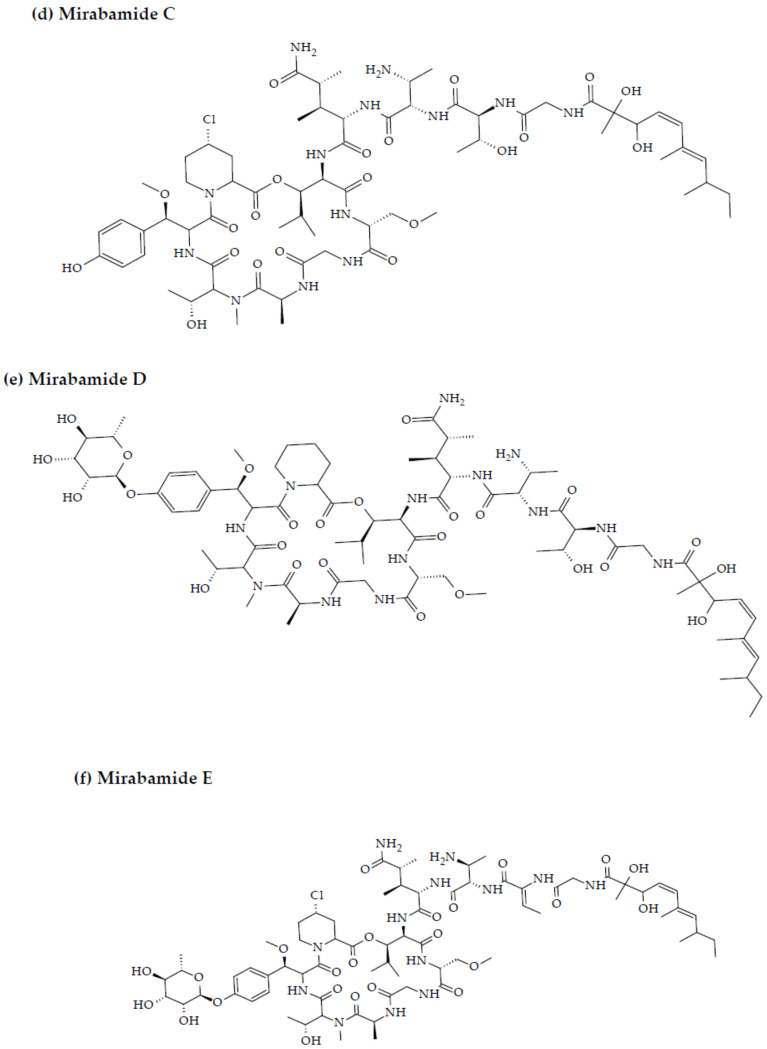

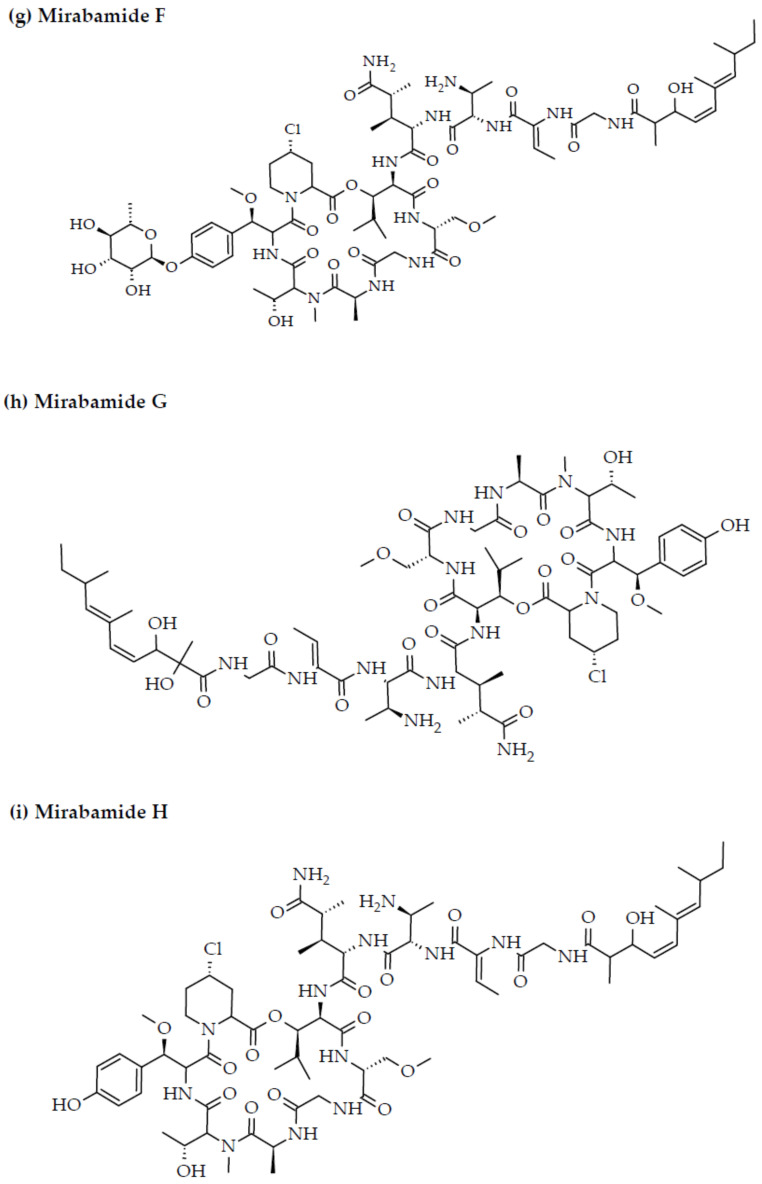

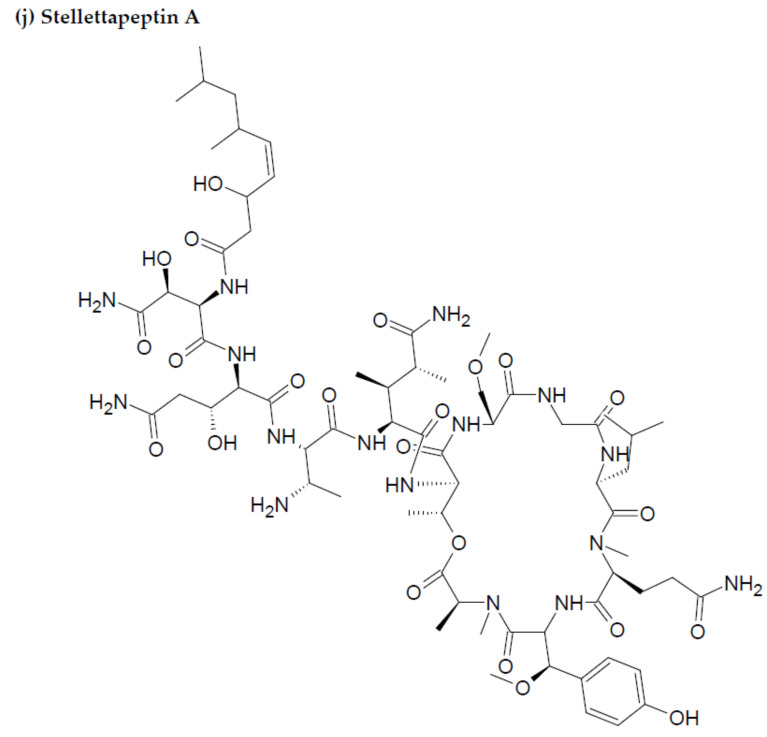

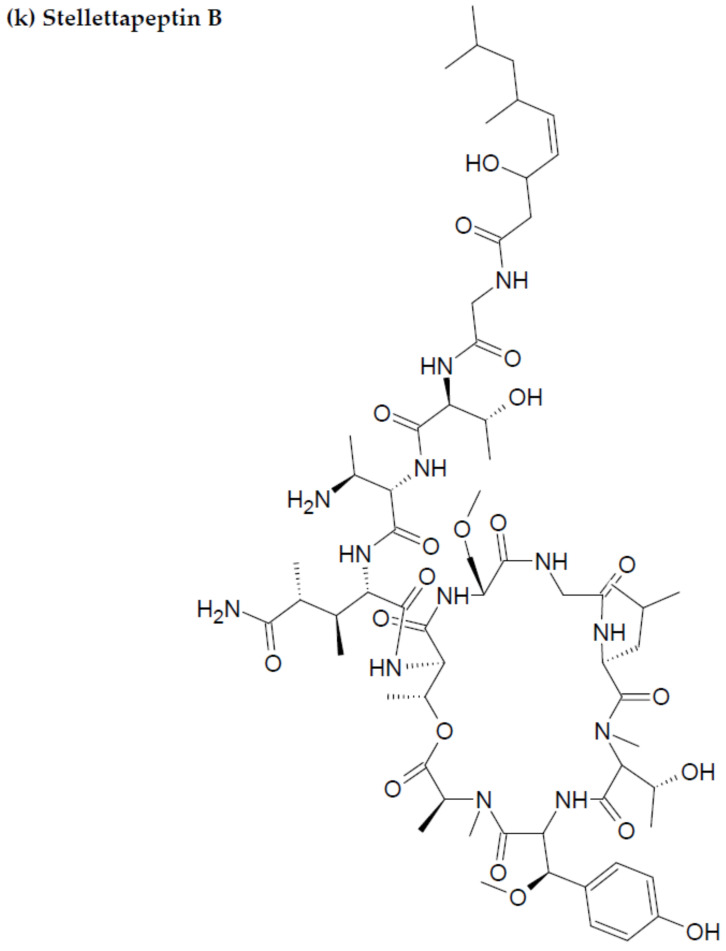

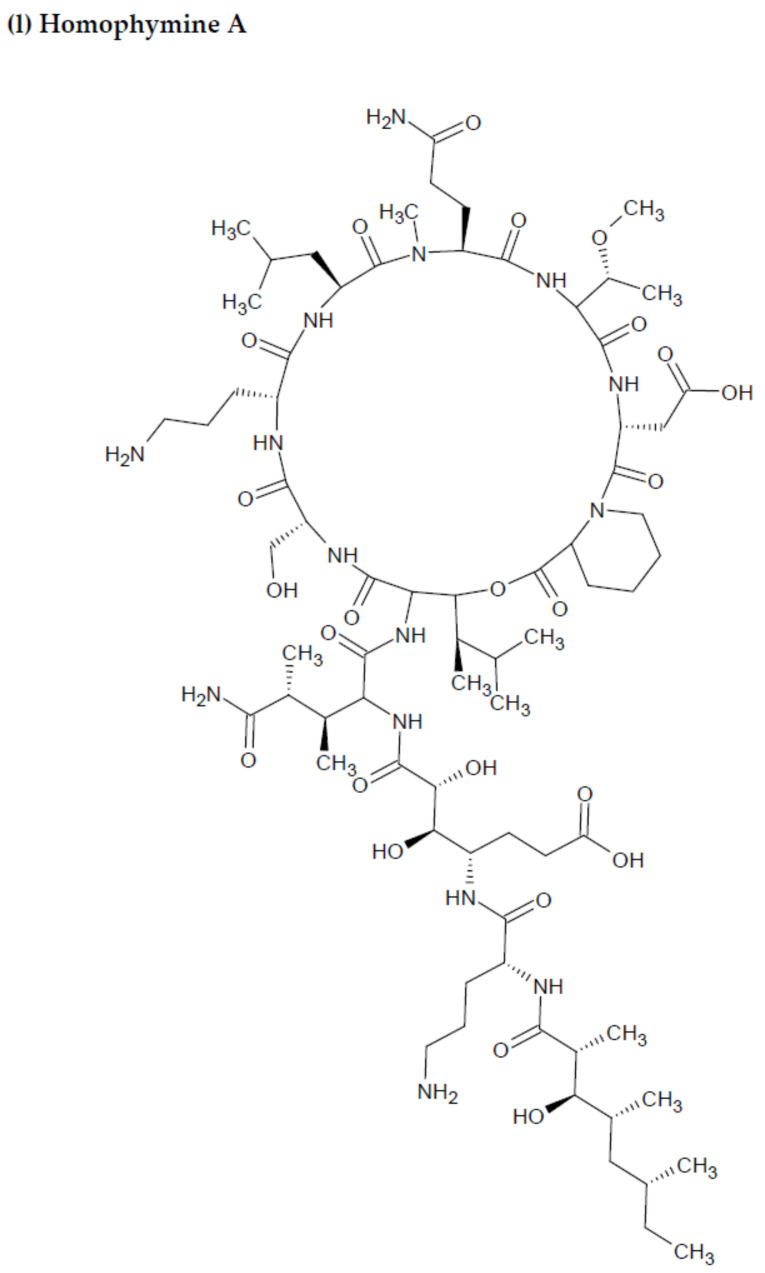

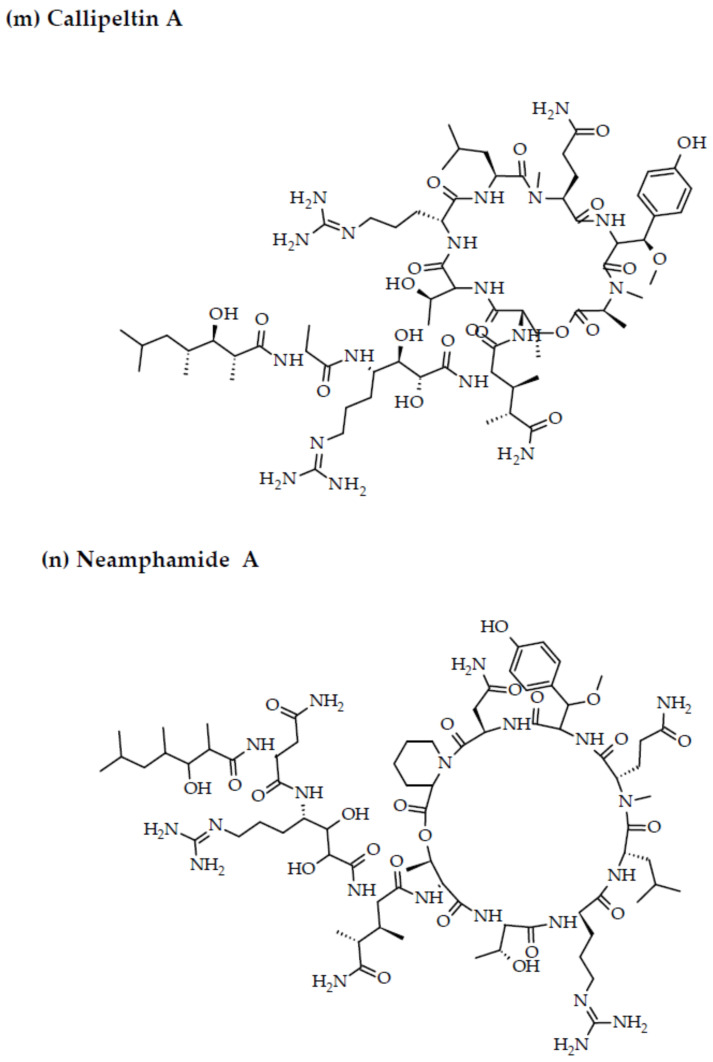

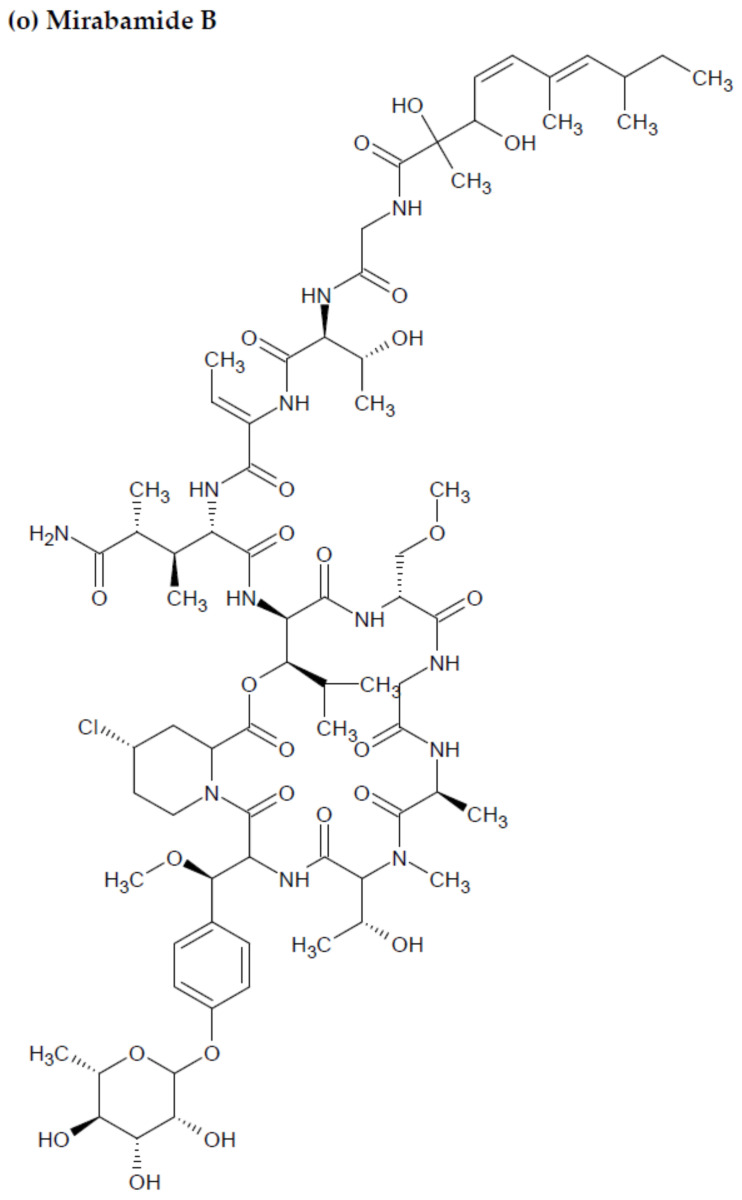
Chemical structures of MBAPs derived from marine sponges. (**a**) koshikamide F, (**b**) koshikamide H, (**c**) mirabamide A, (**d**) mirabamide C, (**e**) mirabamide D, (**f**) mirabamide E, (**g**) mirabamide F, (**h**) mirabamide G, (**i**) mirabamide H, (**j**) stellettapeptin A, (**k**) stellettapeptin B, (**l**) homophymine A, (**m**) callipeltin A, (**n**) neamphamide A, and (**o**) mirabamide B.

One of the primary reservoirs of latent infection is the CD4+T cell containing integrated and transcriptionally silenced HIV-1 proviruses [102]. The activation of these resting cells, particularly after prolonged periods of dormancy, results in proviral gene expression of HIV-1 and consequent renewal of viral infection [103]. The increasing emphasis has therefore been on exploring therapeutic interventions aimed at complete elimination of the latent proviruses (sterilized cure) [104]. Of particular research interest is what is commonly termed as the ‘shock and kill’ strategies, whereby the transcription of HIV-1 proviruses present in resting CD4+ T reservoir cells is de-repressed with the aid of small molecule drugs for the production of replicating virus, which could be inhibited through simultaneous administration of highly active antiretroviral therapy (HAART) [105]. The identification of various latency reversal agents (LRAs) capable of activating HIV-1 proviral gene expression from marine sponge *Phorbas sp*. has been a significant development in this context [103]. The latency reversal activity in the majority of LRAs can be attributed to their ability to activate adenosine 3′,5′-monophosphate (AMP), and protein kinase C (PKC) signaling [106]. The structures of sponge-derived peptides are demonstrated in Figure 3.

### 2.3. Marine Cyanobacteria-Associated Compounds with Anti-HIV Properties

Cyanobacteria are photosynthetic bacteria abundantly observed in nature [107]. They contain a large variety of toxins along with several bioactive compounds with potential bioactive attributes such as antitumor, anticancer, antimicrobial, antifungal, anti-inflammatory, and protease inhibition characteristics.

Cyanovirin-N (CV-N), (Figure 3) an 11 kDa protein derived from the cyanobacteria *Nostoc ellipsosporum* has been tested as an anti-HIV compound (preclinical development) [108]. The IC_50_/EC_50_ values for native CV-N against various HIV-1 isolates range between 0.1 and 36.8 nM, while against HIV-2 isolates, the IC_50_/EC_50_ values range between 2.3 and 7.6 nM [109,110]. In a recent research study, all three forms (mixed, dimer, monomer) of a recombinant version of CV-N (rCV-N) (monomeric mass: 11.962 kDa), exhibited significant anti-HIV activity with an IC_50_ range of 0.5−5 nM, a therapeutic index (TI) of ~1000–10,000 in vitro, negligible cytotoxicity of approximately 5 μM, and low endotoxins (mixed form refers to a mixture of monomer, dimer and higher-order oligomers together). TZM-bl cells were infected with HIV-1_NL4-3_ for the assessment of anti-HIV activity of the rCV-N forms, while their cytotoxicity was determined by an MTT cell proliferation assay. This anti-HIV activity can be attributed to the capability of CV-N to interfere with the fusion of HIV with the CD4+ cell membrane [111].

CV-N has demonstrated specific and strong interaction capabilities with the viral envelop glycoprotein gp120 through *N*-linked oligosaccharides with high mannose content, particularly the glycans Man-8 and Man-9 [112]. This consequently prevents the binding of the gp120 to the host CD4 T-cell receptor, in addition to the chemokine CCR5 and CXCR4 co-receptors, thereby inhibiting both the viral entry and the cell-to-cell fusion and transmission [113]. Owing to its excellent stability, lower toxicity, broad-spectrum antiviral activities, and highly specific binding to oligosaccharides, CV-N has the potential to be a class-leading compound for the prevention of sexual transmission of HIV [114].

Similarly, microvirin (MV-N), isolated from the bloom-forming cyanobacterium *Microcystis aeruginosa*, is another compound with anti-HIV potential [80]. MV-N is a 14.3 kDa protein, and has high specificity for α(1-2)mannobiose at the termini of branched high mannose-type glycans on the viral surface [81]. MV-N, like CV-N, can neutralize a broad range of clinical isolates, and laboratory-adapted strains with low nanomolar potency against most HIV-1 group M clades of various subtypes and tropics [110] with IC_50_ values ranging between 2.1 and 167 nM. MV-N was much more active against HIV-1 isolates A and B when compared to CV-N [115]. MV-N, therefore, is active in the reduction of initiation markers, including CD25, CD69, and HLA-DR, preventing the formation of syncytium between cells infected with HIV-1, and healthy CD4+ T cells, also inhibiting viral replication [115,116]. Furthermore, as the research study by Huskens et al. evidenced, MV-N induced negligible cytotoxicity in MT4 and peripheral mononuclear blood cells (PMBCs) (CC_50_ > 35 μM and CC_50_ > 7 μM, respectively) compared to a much higher CC_50_ value in the case of CV-N (CC_50_ of 191 nM and 900 nM in MT-4 cells and PBMCs, respectively) [115]. It can be inferred, therefore, that MV-N has a greater potency against HIV-1 and a higher safety profile when compared to CV-N.

**Figure 4:**
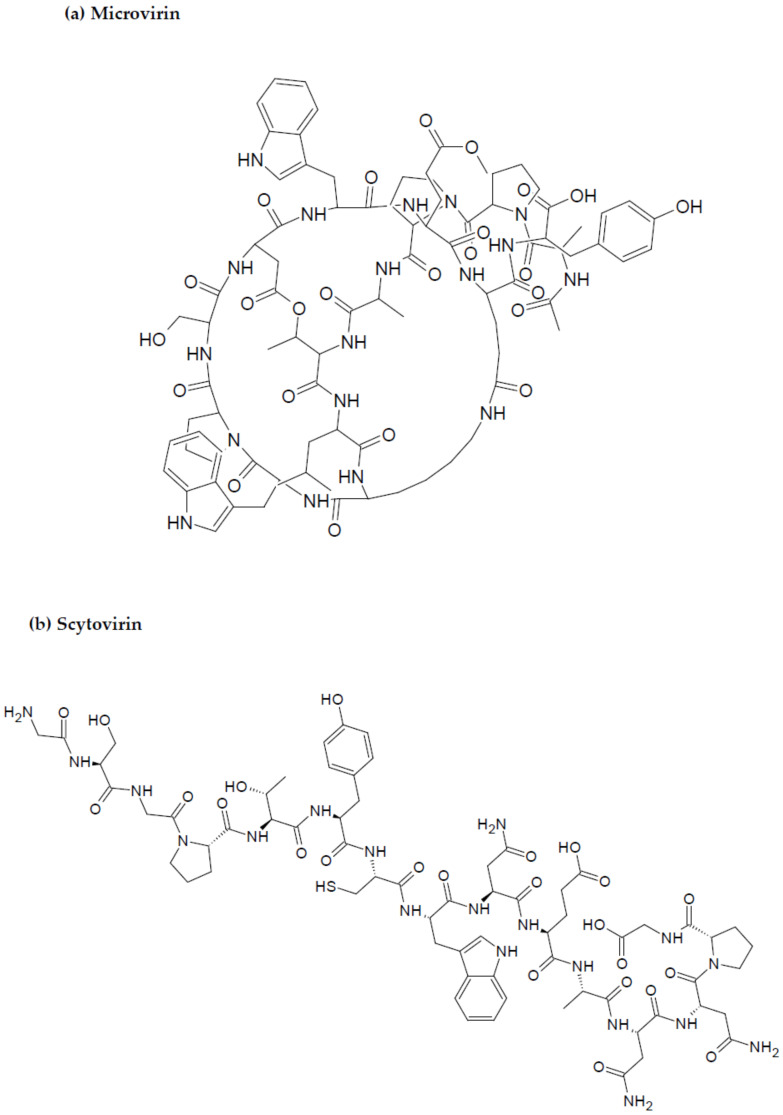
Chemical structures of MBAPs derived from marine cyanobacteria. (**a**) microvirin, and (**b**) scytovirin.

Scytovirin (SV-N) is a 9.71 kDa HIV-neutralizing protein initially isolated from the aqueous extracts of the cultured cyanobacterium, *Scytonema varium* [117]. The compound has a high affinity for mannose-rich oligosaccharides on the gp120 envelope, inducing blockage of the viral entry into the target cell [118,119]. Potent activity has been recorded for SV-N against various HIV-1 clinical isolates and laboratory strains, with EC50 values ranging between 0.4 and 394 nM, and 0.3 and 7 nM, respectively [120]. Recombinant SV-N (rSV-N) also inhibited cytopathicity induced by HIV in side-by-side in vitro XTT-based anti-HIV assays using CEM-SS host cells with an EC_50_ of 4.5 nM, while native SV-N EC_50_ values ranged between 0.3 and 7 nM [120,121]. The structures of the most active cyanobacteria-derived peptides are portrayed in Figure 4.

### 2.4. Marine Seaweed Originated Bioactive Peptides with Anti-HIV Properties

Seaweeds are a sustainable food resource, rich in proteins, carbohydrates, lipids, various pigments, phenolic compounds, minerals, and vitamins [122]. Marine algae have been widely utilized as a food source for centuries, owing to their functional benefits, both as components of mainstream cuisine (seaweeds e.g., kelps, laver/nori), and as hydrocolloidal food thickening additives (carrageenans, alginates, agars) in the food and beverage industries [123]. Algae are chlorophyll-rich organisms, with a worldwide distribution as both unicellular microalgae (microphytes), as well as multicellular macroalgae (seaweeds) bioforms [124]. Their potential as a bioactive source, however, remains largely untapped.

Lectins as a class of glycoproteins, and di- or polyvalent carbohydrate-binding agents (also called hemagglutinins), are significant in the context of mediating host-pathogen interactions, cell–cell communications [110], intracellular signaling cascades, and cellular development [125,126]. Lectins have been proven effective in binding HIV-1 gp120 to the CD4+ host receptors, in addition to being reportedly effective in inhibiting the glycosylation of the HIV-1 protein [127]. Various lectins derived from macroalgae have been reported to have bioactive activities, such as antinociceptive, anti-inflammatory, antibiotic, anti-HIV, and antiplatelet activities [128]. Furthermore, lectins have the potential to inhibit the virion infection of HIV-1 toward the host CD4+ T-lymphocyte cells by interacting with HIV envelope glycoproteins gp120 and gp41 and binding the CD4+, CXCR4, or CCR5 receptors [129,130].

Griffithsin (G-FN), derived from the red algae, *Griffithsia* sp., has emerged as a significant HIV inhibitor [131] with a nanomolar to subnanomolar efficacy (EC_50_ = 0.043–0.63 nM) against a wide range of HIV-1 strains for both native and recombinant G-FN [132,133]. Another promising attribute of G-FN is its broad antiviral spectrum, in addition to its low in vivo and in vitro host toxicity [134]. Because G-FN did not induce mitogenic stimulation of PMBCs exposed to it and exhibited a good safety profile in the rabbit vaginal irritation model, the test is generally regarded as the gold standard for preclinical safety tests for vaginal products. Furthermore, a study conducted to investigate if G-FN exposure elicited any molecular responses in cultured human cervicovaginal cells and PMBCs further validated the safe nature of the drug. An MTT assay indicated no loss of cell viability in ectocervical and endocervical cell lines 3 days post-exposure to up to 1 mg/mL (84 µM) of G-FN, a concentration 10-fold higher than a standard microbicide formulation [135]. Another study also indicated that there was no evidence of direct cytotoxicity in the uninfected control cells upon exposure to the highest tested concentrations of G-FN (783 nM) [133].

Consequently, G-FN is promising for its potential as a general microbicidal agent aimed at the prevention of viral transmission [136]. Furthermore, owing to its specificity for high-mannose arrays present on various pathogenic enveloped viruses, G-FN can be utilized as an effective therapeutic agent in the treatment of diseases mediated by a wide variety of enveloped viruses [137]. Also, G-FN has demonstrated synergistic affinity with many of the currently available HIV-1 antiretroviral drugs, including maraviroc, tenofovir, enfuvirtide, and the broadly neutralizing antibody (bNAb) VRC01 [134].

The green algae *Oscillatoria agardhii* has been a source of *Oscillatoria agardhii* agglutinin homolog (OAAH) proteins, primarily OA agglutinins (OAAs) and hybrid OAAH proteins (OPAs), which have demonstrated activity against HIV-1_NL4.3_ in MT-4 cells with IC_50_ values of 24 ± 5 and 18 ± 2 nM, respectively. Furthermore, the OAAs and OPAs also exhibited inhibitory activity in PBMCs against X4 HIV-1 laboratory strains IIIB and NL4.3 (proviral clone R9) (IC_50_ values of ~30 and 7.5 nM, respectively), in addition to R5 and X4/R5 viral strains, and various subtype HIV-1 isolates (groups M and O) (IC_50_ values ranging between 6 and 57 nM for OAAs and OPAs, respectively). Overall, OAAs and OPAs exhibited activity against the HIV-1 laboratory strains irrespective of the coreceptor used (IC_50_ value ranges 19–60 nM and 8–31 nM, respectively) [138].

Green algae *Boodlea coacta* (BCA) also contains lectins with a high potential of inhibiting HIV-1 (EC_50_ of 8.2 nM), with high specificity for α1-2 linked mannose at the non-reducing terminus [139]. Similarly, the lectin ESA-2, a purified agglutinin obtained from red algae, *Eucheuma serra*, exhibits unique specificity and a preferential affinity for high mannose N-glycans, and demonstrated activity against HIV-1 X4 strains in MT-4 cells with EC_50_ of 165 nM [140].

### 2.5. Marine Mussels, Ascidians, and Oyster-Derived Bioactive Peptides against HIV

**Figure 5:**
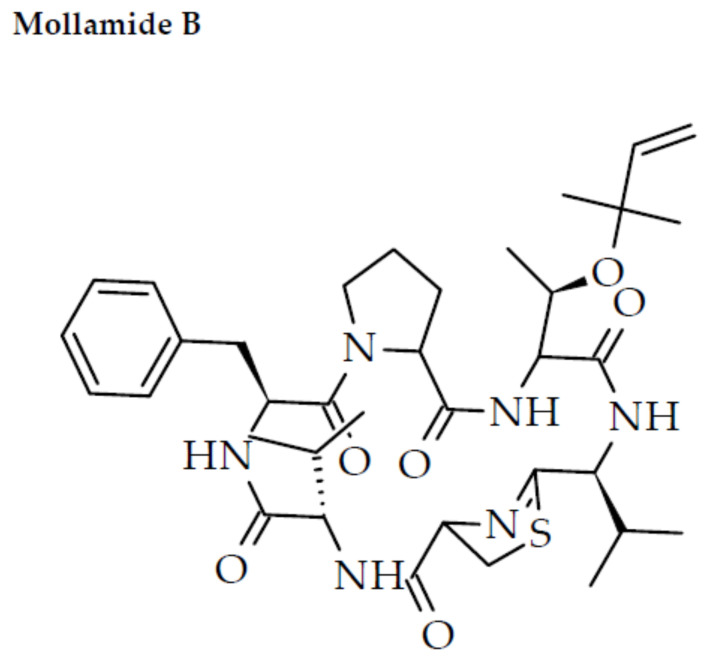
Chemical structure of mollamide B derived from ascidians.

Human consumption of mussels has witnessed an increasing trend in recent times owing to their health-promoting biological attributes [141]. The cyclic and linear peptides derived from marine ascidians have been found with anti-HIV potential with many other biological properties. A cyclic heptapeptide, mollamide B (Figure 5), isolated from the colonial tunicate *Didemnum molle* indicated cytotoxicity against HIV-1 in human PBMCs at an EC_50_ value of 48.7 μM [142].

Two peptides, P1 and P2 [sequences LLEYSI (P1), and LLEYSL (P2)] have been isolated from the oyster (*Crassostrea gigas*) proteins hydrolysate prepared with thermolysin and have exhibited competitive HIV-1 protease inhibition activity (IC_50_ values of 20 and 15 nM, respectively, and *Ki* values of 13 and 10 nM, respectively) [143,144,145]. Clavanin B (peptide sequence: VFQFLGRIIHHVGNFVHGFSHVF), an alpha-helical peptide isolated from the tunicate *Styela clava* also exhibited anti-HIV activity with an EC_50_ value of 7.1 μM, TC_50_ value of 37.1 μM, and TI of >5.18 [146,147].

### 2.6. Anti-HIV Bioactive Peptides Isolated from Marine Bacteria and Fungi

The symbiont microorganisms associated with various marine organisms have been cited as synthesizers of various bioactive molecules, and therefore, sources of these highly significant moieties [55]. Of particular significance among these microorganisms are bacteria (representing as many as 47 phyla) from the bodies of marine sponges, as close to one-third of these have been proven to be the producers of bioactive peptides [148]. However, bacteria isolated from other marine organisms such as algae, plants, cnidarians, and mollusks have also proven to be valuable prospects for the isolation of bioactive compounds [149]. The anti-HIV therapeutic isolates from bacteria include compounds from classes such as polyketides, terpenes, peptides, and some lactones, including macrolactins A-F, exhibiting high anti-HIV potential [150]. For instance, three hexapeptides, isocomplestatin, chloropeptin II (complestatin), and chloropeptin I, have been isolated from *Streptomyces* sp., and are notable for their anti-HIV potential. In this regard, isocomplestatin exhibited inhibitory action in coupled 3′-end processing/strand transfer (IC_50_: 200 nM), strand transfer (IC_50_: 4 µM), and HIV-1 replication (IC_50_: 200 nM) in virus-infected cells. Similarly, isocomplestatin also exhibited inhibitory activity against the strand transfer of recombinant integrase in staged assays and integration (using isolated HIV-1 preintegration complexes) with IC_50_ values of 3–4 µM, in addition to HIV-1 replication in cell culture (IC_50_ of 0.2 µM). Both chloropeptin I and complestatin (or chloropeptin II) are inhibitors of gp120-CD4 binding (IC_50_: 2–1.3 µM). With regard to HIV-1 integrase activity, complestatin A, chloropeptin, and complestatin B, although less active than isocomplestatin, demonstrated their anti-HIV potential in the coupled and strand transfer assays with IC_50_ values of 0.4–1.7 and 5–12.5 µM (with reference to the assays), respectively [151,152].

**Figure 6:**
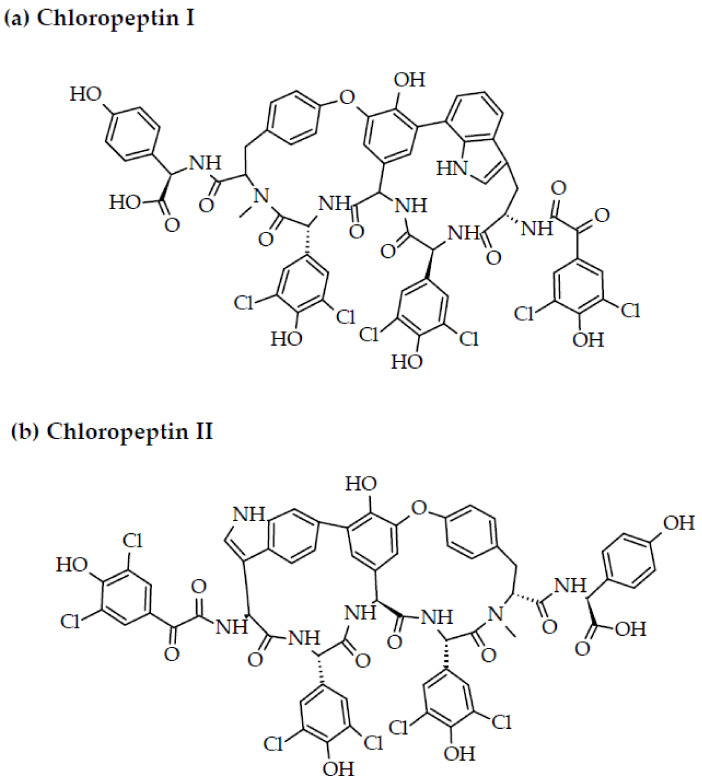

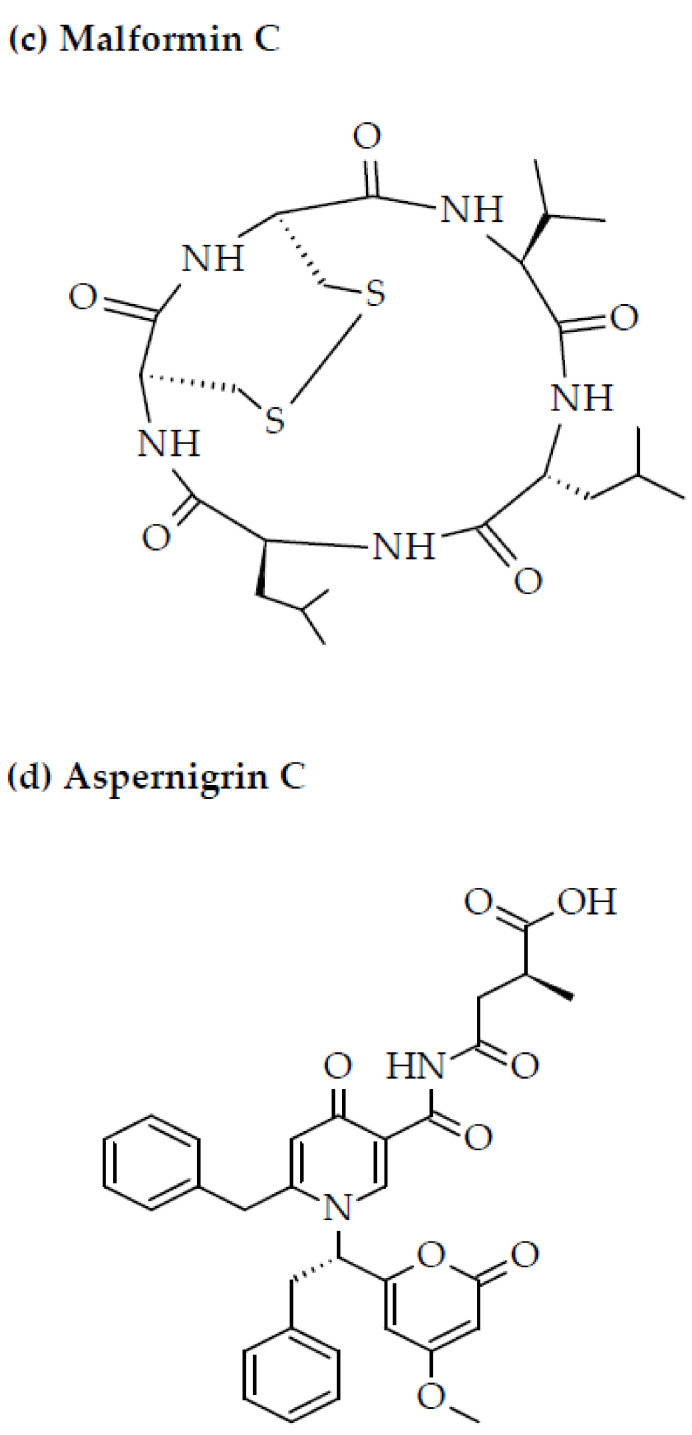

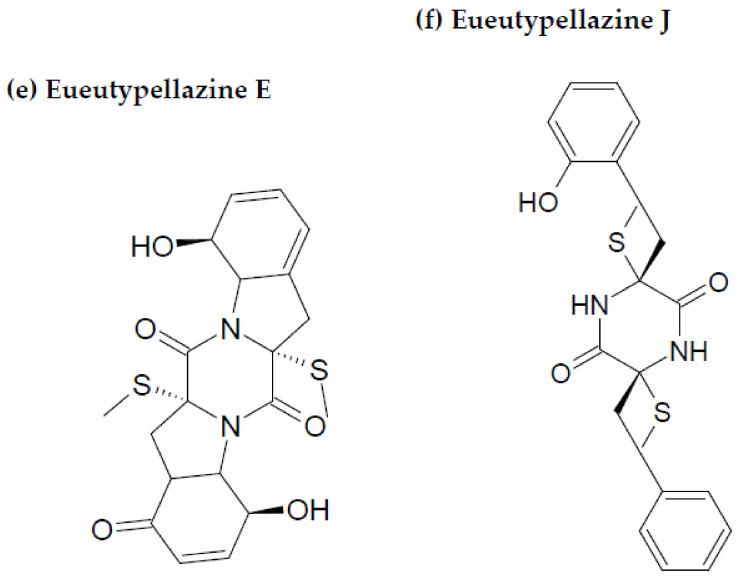
Chemical structures of MBAPs derived from marine bacteria and fungi. (**a**) chloropeptin I, (**b**) chloropeptin II, (**c**) malformin C, (**d**) aspernigrin C, (**e**) eutypellazine E, and (**f**) eutypellazine J.

*Aspergillus niger* SCSIO Jcsw6F30, commonly associated with the marine brown macroalgae *Sargassum* sp., has been the source of malformin C, a cyclic pentapeptide, and aspernigrin C, which have exhibited anti-HIV potential with IC_50_ values of 1.4 (SI: 11.4), and 4.7 µM (SI: 7.5), respectively, against infection by CCR5-tropic HIV-1 SF162 in TZM-bl cells [153]. Eutypellazine E and Eutypellazine J, isolated from the deep-sea sediment fungus *Eutypella* sp. MCCC 3A00281, are also notable for their inhibitory effects against HIV-1 replication (IC_50_ values of 3.2 and 4.9 µM) in pNL4.3.Env-.Luc co-transfected 293T cells [153]. Eutypellazine J also demonstrated inhibitory action against HIV-1 latency-reactivating agents with an IC_50_ value of 80 µM, and low cytotoxicity at CC_50_ > 100 μM in J-Lat A2 cells [153,154]. These compounds, therefore, can be utilized as base materials for the treatment of HIV. The structures of bacteria and fungi-derived peptides are portrayed in Figure 6.

## 3. Processing Techniques for Marine Bioactive Peptides

MBAPs vary based on the species they are being isolated from, in addition to the amino acid composition, amino acid sequencing, structure, and conformations; therefore, they can be prepared by utilizing various techniques [55]. Salient requisite characteristics for such a processing technique include industrial scalability, lower operational costs, biocompatibility, and reproducibility [64]. Enzymatic hydrolysis, induced by digestive enzymes, remains one of the primary methods for producing various marine BAPs embedded and encrypted in the structures of mature parental proteins [14]. Similarly, microbial fermentation is another methodology that employs bacterial hydrolases for breaking down proteins into smaller peptide molecules [55].

Different techniques have been formulated to extract and purify the bioactive peptides from various sources. Organic synthesis is one of the many techniques being progressively applied to marine products, which employs solvents and mass spectrometry to recognize the coarse product. However, this technique is more time-consuming, less environmentally friendly, costly, and requires a clear sequence of peptides [155]. Peptides are also extracted through a microwave extraction technique that utilizes electromagnetic radiations of 300 MHz to 200 GHz to heat solvent and isolate the component of interest [156]. It has been applied to oysters, fish tissues, and shrimps for the extraction of bioactive peptides [157,158]. The technique utilizes less solvent, has lower energy needs, has better yield, is more environment-friendly, and is cost-effective as compared to traditional extraction techniques. However, a high-temperature application may degrade the bioactive compounds [14,159].

Chemical synthesis of MBAPs involves their production from amino acid units in a pre-defined environment (e.g., a solid medium), primarily by way of two major pathways, soluble-phase, and solid-phase syntheses, and sometimes, a third pathway, known as the hybrid synthesis, which is usually employed to produce pharmaceutical peptides of critical significance [160]. The major advantage associated with these methodologies is their cost-effective nature [55]. However, they do present some limitations in the form of undesirable synthesis of intermediates, long processing time, the insolubility of long peptide chains in organic solvents, generation of chemical waste, difficulty to control the process, generation of altered amino acids, and variability in structural and functional properties [161,162].

Acid hydrolysis has found its applications in extraction from fish scales, mackerel, salmon, and catfish [14]. Moreover, enzymatic hydrolysis has also been successfully applied to extract bioactive peptides from certain seaweeds [60]. Better control, reproducibility, and maintenance of nutritional content are some of the core benefits associated with this technique. Bioactive peptides extracted from different marine sources, mainly seaweeds BAPs (red, brown, and green algae), are further purified through membrane filtration, high-performance liquid chromatography, ion-exchange chromatography, and gel filtration chromatography [24].

Recent extraction approaches for marine seaweed and marine mussels include microwave-assisted extraction, supercritical fluid extraction, pressurized solvent extraction, ultrasound-assisted extraction, pulsed electric field extraction, and various enzyme-assisted extraction techniques [14,60]. Extraction is followed by hydrolysis to facilitate the release of the peptide fragment [163]. The extraction and purification techniques commonly employed in this regard are outlined in Table 1. Rawendra [163] isolated the peptides (lysozyme-derived peptide, IW-11) from soft-shelled turtle (*Pelodiscus sinensis*) egg white using ultrafiltration and reversed-phase high-performance liquid chromatography (HPLC). Moreover, proteolysis in combination with fermentation and enzymatic hydrolysis also extracts short-chain peptides [49].

Recombinant production is another recent development that involves the production of BAPs, whereby the peptide genes are permitted to be expressed in a specific expression system, either in vivo or in vitro (also called the cell-free system) [14,55]. Major advantages of this method include the high operation speed, and that it allows for the mass production of the desired peptides. However, high operation costs remain a veritable constraint, preventing mass industrial application and utilization [160,161].

## 4. Conclusions, Limitations, and Future Perspectives

The discovery of the MBAPs with anti-HIV properties has opened a new horizon for the drug and pharmaceutical industry, and therefore, MBAPs have extensively been the subject of the latest research. The limitation of using MBAPs as drugs can be attributed to limited research on drug delivery action, and the lack of available harmonized research data related to the extraction of MBAPs and their derivatives.

To use these MBAPs as a therapeutic agent against HIV, the chemical structure, amino acid profile, exact length of peptides, ratio of peptides that can easily pass through the human gastrointestinal tract, and pathway for release at the target site must be known. However, the data is not sufficient in this regard, with only a few clinical and non-clinical trials being reported thus far. Nonetheless, to use these MBAPs on a commercial scale as an anti-HIV drug, more research and clinical studies are required to produce readily available, easy-to-use, and clinically safe products. For using the BAPs from marine sources, it is essential to consider that they may undergo chemical modifications, ultimately losing their activity during processing. Past studies have reported that the harsh environmental conditions during processing cause various modifications in the chemical structures of the MBAPs [164]. Encapsulation and nanoencapsulation can be highly viable and promising techniques for producing MBAP-based drugs, and for overcoming processing-associated problems (pH, temperature, overheating, and exposure to oxygen), thereby ensuring their greater bioavailability and bioaccessibility, in addition to improved efficacy [165].

To strengthen their industrial application, the consumption pattern of MBAPs, the mode of action on the cell cycle, and apoptosis must be profoundly researched. Conversely, in the future, omic technologies (genomics, proteomics, and metabolomics) can be used to discover more novel varieties of BAPs with the potential to be used as anti-HIV drugs, both in blended form and individually. An increasing number of in vitro studies have reported various purification techniques (membrane filtration, gel filtration chromatography, HPLC, and ion exchange technique) for MBAPs (consisting mostly of between 3–30 amino acid units). Despite significant improvements, the low yield and high installation costs associated with the utilization of these techniques remain a major impediment in the context of their application on an industrial and commercial scale. This study can also be expanded in the future by exploring new, cost-efficient, high-yield-producing purification techniques for their use in omics studies. The discovery of MBAPs effective for the treatment of HIV has opened many research domains for future studies.

## Figures and Tables

**Table 1:** Summary of the techniques used for the preparation and purification of marine bioactive peptides commercially [72,73,74,75,76,77,78,79,80,81,82,83,84,85].

Techniques	General Properties	Type of Peptides	Sources	Solvents/ Chemicals Applied
Preparation Techniques				
Solvent extraction	Less effective and time-consuming	Collagen peptides	Cod skin, marine crab, hemolymph	Ethanol, methanol, acetone, ethyl acetate, hexane butanol, and methanol
Microwave-assisted extraction (MAE)	Uses electromagnetic radiations of 300 MHz to 300 GHz, enhanced yield, rapid and selective extraction		Applied to fish, shrimp, brown seaweed, and oyster	Utilizes a series of solvents from heptane to water
Chemical Hydrolysis	Simple and inexpensive	Fish protein hydrolysates	Applied to fish, bycatch fish	Sulfuric acid, hydrochloric acid, nitric acid, malic acid, oxalic acid, and phosphoric acids are majorly used
Enzyme Hydrolysis	Alcalase, flavourzyme, neutrase, trypsin, pepsin, papain, and bromelain are primarily used, more controllable and reproducible method, temperature, time, pH, enzyme concentration, and water/matter ratio are the major variables	Two purified dipeptides Tyr-Arg (337.2 Da) and Ile-Arg (287.2 Da), crude protein hydrolysates, papain hydrolysates, YVMRF peptide	Marine algae, marine sponge (*Stylotella aurantium*), *Bellerochea* and *Nitzschia* species, *Stolephorus chinensis*	
Increased cost and fouling are the main problems, MF (pore size is 0.1–10 µm), UF (pore size is 0.001–0.1 µm)		UF is used for nonhydrolyzed proteins and macro peptides	Generally applied to mackerel, shrimp, *Spirulina platensis*	
Gel Filtration Chromatography	Simple and mild method, separate based on size, varying elution conditions, higher selectivity, and resolution, sample time consumption is the major limitation		Applied to fish and marine plants	Aqueous buffer (pH 6–8)
Ion-Exchange Chromatography	It captures target protein and bulk impurities, Capto, MacroCap, and Monobeads are some of the mediums used		Applied to the mussel	Aqueous solutions or buffers containing organic solvents such as methanol and acetonitrile
* HPLC including RP-HPLC, MS, LC-MS/MS, ESI, MALDI-TOF, HPLC-ELSD, UHPLC-MS/MS and RRLC–MS.	Higher resolution an sensitivity, ease of operation, rapid, expensive and environment unfriendly	SAITAPGGAM peptide, collagen peptides, cyclic heptapeptide Euryjanicin A, ALGPGPT, LVPPLA, LAPPTM, GVLIG and GHPVL, ILTLAALGGL, IITGGL, AAPSTVL, and TVAPPGA	Applied to cyanobacteria, fish, sponge, snail and *Palmaria palmata*, cod skin, *Prosuberites laughlini,* Stonefish, *Dunaliella salina*	Choline-oxalic acid based on eutectic solvent

* HPLC (High performance liquid chromatography), reverse-phase—high-performance liquid chromatography, RP-HPLC, mass spectrometry (MS), liquid chromatography followed by tandem mass spectrometric detection (LC-MS/MS), electrospray ionization (ESI), matrix-assisted laser desorption/ionization time-of-flight (MALDI-TOF), high-performance liquid chromatography with evaporative light scattering detection (HPLC-ELSD), ultra-high-performance liquid chromatography-tandem mass spectrometry (UHPLC-MS/MS), and liquid chromatography-tandem mass spectrometry (RRLC–MS).

## Data Availability

Not applicable.
